# Association between Self-Reported Gluten Avoidance and Irritable Bowel Syndrome: Findings of the NutriNet-Santé Study

**DOI:** 10.3390/nu13114147

**Published:** 2021-11-19

**Authors:** Anouk Reuzé, Rosalie Delvert, Laëtitia Perrin, Robert Benamouzig, Jean-Marc Sabaté, Michel Bouchoucha, Benjamin Allès, Mathilde Touvier, Serge Hercberg, Chantal Julia, Emmanuelle Kesse-Guyot

**Affiliations:** 1 Nutritional Epidemiology Research Team (EREN), Sorbonne Paris Cité Epidemiology and Statistics Research Center (CRESS), Inserm U1153, Inrae U1125, Cnam, Université Sorbonne Paris Nord University, 93017 Bobigny, France; rosalie.delvert@agroparistech.fr (R.D.); laetitia.perrin@inserm.fr (L.P.); b.alles@eren.smbh.univ-paris13.fr (B.A.); m.touvier@eren.smbh.univ-paris13.fr (M.T.); s.hercberg@uren.smbh.univ-paris13.fr (S.H.); c.julia@uren.smbh.univ-paris13.fr (C.J.); e.kesse@eren.smbh.univ-paris13.fr (E.K.-G.); 2 Department of Hepatology and Gastroenterology, Avicenne Hospital (AP-HP), 93017 Bobigny, France; robert.benamouzig@aphp.fr (R.B.); jean-marc.sabate@aphp.fr (J.-M.S.); michel.bouchoucha@aphp.fr (M.B.); 3 Physiopathologie et Pharmacologie Clinique de la Douleur, Ambroise Paré Hospital, 92104 Boulogne Billancourt, France; 4 Department of Public Health, Avicenne Hospital (AP-HP), 93017 Bobigny, France

**Keywords:** irritable bowel syndrome, functional gastrointestinal disorder, self-management, dietary exclusion, gluten-free diet

## Abstract

Self-management of irritable bowel syndrome (IBS) is increasingly focusing on exclusion diets. In particular; patients are showing a significant interest in the gluten-free diet for the treatment of IBS. However; the lack of scientific evidence prevents the establishment of clear dietary guidelines and attention is needed as dietary restriction can lead to potentially adverse effects. This cross-sectional study aims to explore the practice of gluten avoidance in participants identified with IBS in a large cohort of non-celiac French adults. The population included 15,103 participants of the NutriNet-Santé study who completed a functional gastrointestinal disorder questionnaire based on the Rome III criteria to identify IBS in 2013 and a food avoidance questionnaire in 2016. Data on diet and anthropometric and sociodemographic characteristics were collected. Multivariate logistic regression models were used to compare the avoidance of gluten between IBS and non-IBS participants. Participants were mainly women (73.4%) and the mean age in this population was 55.8 ± 13.2 years. Among these individuals, 804 (5.4%) participants were identified as IBS cases. Among them, the prevalence of gluten avoidance was estimated at 14.8%, of which 3.0% reported total avoidance; versus 8.8% and 1.6% in non-IBS participants. After adjustments; gluten avoidance was higher in IBS participants compared to their non-IBS counterparts: (OR = 1.86; 95%CI = 1.21, 2.85) for total and (OR = 1.71; 95%CI = 1.36, 2.14) for partial avoidance. Participants identified with IBS were more associated with gluten avoidance than non-IBS participants. Further studies are needed to explore the long-term consequences of dietary interventions and to provide consistent dietary guidance connected to patient perception.

## 1. Introduction

Irritable bowel syndrome (IBS) is a functional gastrointestinal disorder (FGID) characterized by abdominal pain or discomfort associated with altered bowel habits while abnormalities of the gastrointestinal tract have been ruled out [1]. The prevalence of IBS is estimated to be approximately 11% and occurs more frequently in women and young adults [2]. Due to the lack of reliable biomarkers, diagnosis is established on a clinical evaluation of symptoms and patients are classified according to four patterns based on the predominant gastrointestinal symptoms as described by the Roma III criteria: IBS with predominant constipation (IBS-C), IBS with predominant diarrhea (IBS-D), IBS with predominant irregular bowel habits (mixed C/D) (IBS-M), IBS unclassified (IBS-U) [3]. Physiopathology of IBS is complex, multifactorial, and unspecific, which still leads to considerable uncertainty [1], and active pharmacological treatments are still lacking [4]. However, diet has emerged among patients as an efficient approach to the therapeutic management of IBS [5]. Meal ingestion is commonly identified as worsening gastrointestinal (GI) symptoms in IBS patients [6], and certain foods groups or specific foods are reported to contribute to symptoms: wheat-based food, dairy food, alcohol, caffeine, fatty, and spicy food but also vegetables and some fruits [7,8]. Specific dietary interventions were subsequently studied in the management of IBS, including dietary fiber, low-fat diet, lactose-free diet, gluten-free diet [9].

Found in wheat, barley, and rye, gluten is ubiquitous in the Western diet but now, cutting gluten from the diet has become increasingly popular. The high demand for gluten-free products reveals the widespread avoidance of gluten among some segments of the population [10,11]. Common reasons for excluding gluten are the perception of a healthier diet, apart from the improvement of gastrointestinal symptoms [12]. In recent years, gluten-related disorders have drawn attention and have been described in a wide spectrum of gluten reactions. While celiac disease and wheat allergy require a strict withdrawal of gluten, both affect only 1% of the general population [13]. In addition, many so-called symptomatic gluten avoiders still complain of GI disorders when ingesting gluten-containing products as documented in our previous study [14].

Over the past 10 years, clinical research on IBS has focused on the gluten-free diet. Several studies have shown improvement in GI symptoms after gluten exclusion in a subset of IBS patients [15], but yet, evidence on the efficacity of gluten avoidance is still debated within the scientific community, hindering the assertion of clear and consistent directions. In the meantime, the practice of gluten avoidance appears to be highly approved by IBS patients as many have tried or are still following a gluten-free diet [16,17]. Along with gluten, other wheat components showed to be potential symptom inducers: amylase/trypsin inhibitors (ATIs), wheat germ agglutinins, fermentable oligosaccharides, disaccharides, monosaccharides, and polyols (FODMAPs). Indeed, a dietary intervention based on a low-FODMAP diet appears to be the preferred dietary option for IBS by gastroenterologists while patients demonstrate a greater interest in gluten avoidance [16].

Currently, a large number of patients with IBS remain undiagnosed and most recent studies have investigated food avoidances among patients clinically diagnosed. This cross-sectional study aims to describe the self-reported practice of gluten avoidance (total or partial) in participants identified with and without IBS among a sample of non-celiac French adults from a large cohort in the NutriNet-Santé study.

## 2. Materials and Methods

### 2.1. Study Population

The NutriNet-Santé is an ongoing, web-based, prospective observational cohort study, launched in 2009. It includes a large population of volunteers aged over 18 years old recruited from the general French population [18]. It aims at investigating the relationship between nutrition and health outcomes and at focusing on determinants of dietary behaviors and nutritional status. At baseline and yearly after that, participants are requested to complete a set of self-administered questionnaires on sociodemographic, lifestyle, health, diet, physical activity, and anthropometric characteristics. Moreover, additional questionnaires are punctually submitted on specific topics related to eating behaviors, nutritional status, and health.

#### Ethics

The NutriNet-Santé study is being conducted in accordance with the Declaration of Helsinki and was approved by the Institute Review Board of the French Institute for Health and Medical Research (00000388FWA00005831) and the Commission Nationale de l’Informatique et des Libertés (CNIL 908450 and 909216). All participants provided electronic informed consent. The cohort study is registered in Clinical Trials.gov (NCT03335644).

### 2.2. Data Collection

#### 2.2.1. Irritable Bowel Syndrome (IBS)

An additional questionnaire sent in 2013 assessed functional gastrointestinal disorders (FGIDs). History of digestive diseases and symptoms were collected using the Rome III questionnaire [3] to define IBS (with minimal symptom duration of at least six months) and IBS subtypes: IBS-C, IBS-D, IBS-M, IBS-U. The questionnaire also perceived other functional diseases (constipation, diarrhea, dyspepsia). Participants reporting organic disease (stomach, esophagus or colorectal cancers, familial adenomatous polyposis coli, Crohn’s disease, celiac disease, ulcerative colitis) or alarm symptoms (melena, hematemesis, rectal bleeding, or significant unintentional weight loss in the past three months) were excluded from the present study.

#### 2.2.2. Gluten Avoidance

From September to December 2016, participants were invited to complete an optional questionnaire assessing food avoidances and their related motives. The questionnaire was divided into three parts: self-reported avoidance of 83 specific types of foods that are often avoided, self-reported specific diets and the related motivations, and self-reported allergies. Regarding gluten, participants were asked the following question: “Do you avoid products containing wheat/barley/rye/oats (gluten) from your diet?”. The responses were “Yes, totally/Yes, partially/No”. Participants were classified into three groups based on self-reported gluten avoidance: total avoiders, partial avoiders, non-avoiders [14]. Similar questions about dairy products were collected to identify individuals avoiding lactose for health reasons only.

#### 2.2.3. Sociodemographic and Lifestyle Data

Individual sociodemographic, anthropometric, and lifestyle characteristics were collected, including sex, age, monthly household income, educational level, occupational category, marital status, size of the urban residence unit and smoking status [19]. Height and weight were also reported using a validated questionnaire [20]. Body mass index (BMI) was calculated as weight (in kg) per squared height (in m^2^), and participants were classified according to WHO standards: underweight, normal, overweight, obesity [21]. Monthly income per consumer unit (c. u.) was calculated on the monthly household wage basis and weighted by the household composition. One c. u. was attributed to the first adult, 0.5 c. u. for other individuals aged over 14 years old and 0.3 c. u. for children under 14 years old [22]. Thus, participants have been divided into different categories: <1200 € per c. u./1200–2300 € per c. u./> 2300 € per c. u./Refuse to declare. Physical activity was assessed using the International Physical Activity Questionnaire (IPAQ) at baseline [23]. Three categories, according to the level of physical activity, were defined. Characteristics were collected as close as possible to the period of completion of the food avoidance questionnaire.

#### 2.2.4. Dietary Data

At baseline and every six months, dietary intakes were collected through web-based, self-administered 24-h dietary records using validated tools [24,25]. The dietary assessment method relies on a meal-based approach, reporting each food and beverage consumed at any eating occasion. Portion size for each item was also estimated using photographs from a validated picture manual [26].

Three non-consecutive-day dietary records were randomly assigned over a two-week period (two week-days and one weekend day) to estimate the intra-variability of the daily intake. Only participants who filled in at least three 24-h dietary records during the two-year period preceding the food avoidance questionnaire were considered in the present study and all their dietary records completed during the 2-year were averaged. Energy, macro and micronutrient values from the dietary questionnaire were estimated by the NutriNet-Santé food composition table, gathering more than 3500 different foods [27]. The simplified *Programme National Nutrition Santé*—Guidelines Score 2 (sPNNS-GS2), which reflects the adherence to the French dietary recommendations, was calculated [28]. Twenty-three food groups were defined for descriptive purposes.

### 2.3. Statistical Analysis

Participants included in the NutriNet-Santé cohort study who completed both food avoidance and FGID questionnaires and who had available dietary data and individual characteristics were considered in the present analysis. Those with organic disease(s) or alarm symptoms, listed above, were excluded from the sample.

A description of sociodemographic, anthropometric, and lifestyle characteristics of the total sample and according to IBS status was performed; p-value referred to chi-square or ANOVA tests according to the type of variable. Motives for gluten avoidance, self-reported food avoidances and particularly for lactose avoidance, self-reported gluten intolerance or sensitivity and self-reported allergies were described according to IBS status. Nutrient intakes were presented according to IBS and non-IBS participants (Table S1). Macronutrients were considered as the percentage of energy intake. Micronutrient intakes were adjusted for energy intake using the residual method [29].

Dietary profiles were extracted using principal component analysis (PCA). PCA was performed on 23 food groups. Factors were identified using Cattel’s scree test and the interpretability of the factors [30]. Associations between food groups and principal components were analysed by correlations and factors loadings. Three independent dietary patterns were identified and used as cofactors in the models: healthy diet, traditional diet, and Western diet (Table S2).

Multivariable multinomial logistic regression models were conducted to assess the association between gluten avoidance and IBS status. Three different models were computed increasing adjustments accounting for known or potential confounders: model 1, adjusted for sex and age (continuous); model 2, adjusted for covariates of model 1 + educational level, monthly household income, occupational category, physical activity, smoking status, total energy intake excluding alcohol (continuous), alcohol (continuous) and the number of dietary records completed since the inclusion (continuous); model 3, adjusted for covariates of model 2 + BMI (continuous). The principal model is model 4, adjusted for covariates of model 3, with further adjustment for sPNNS-GS2 (continuous) (model 4). An additional model (model 5) replacing the sPNNS-GS2 by dietary patterns score derived from PCA was performed (model 5). Association between gluten avoidance and IBS subtypes was also assessed by a binomial logistic regression and adjusted for the same covariates as model 4 A binary outcome was used, gathering total and partial gluten avoidances due to statistical power issues.

All tests were two-sided, and a *p*-value < 0.05 was considered significant. Statistical analyses were conducted with SAS (version 9.4, SAS Institute, Inc., Cary, NC, USA).

## 3. Results

### 3.1. Sample Selection and Description

**Figure 1:**
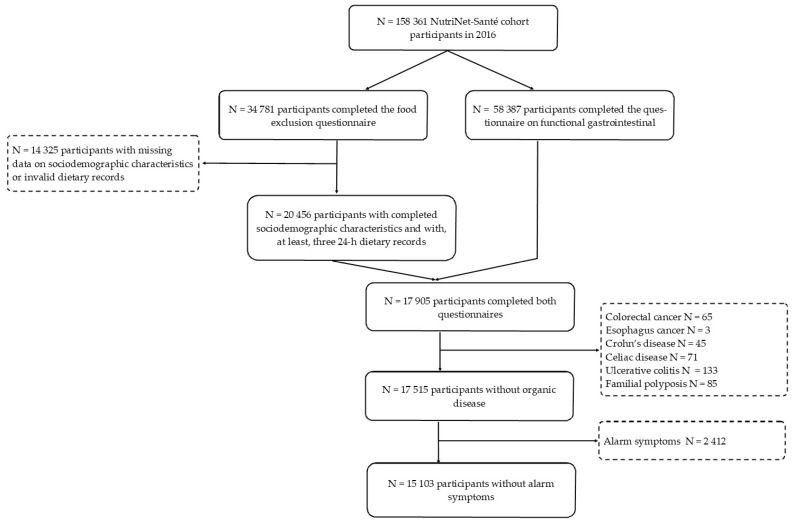
Flowchart of the study.

Among 158,361 participants included in the NutriNet-Santé study in 2016, a total of 34,781 participants filled in the additional food avoidance questionnaire. Among them, after exclusion of missing data for dietary, sociodemographic, and lifestyle characteristics, we selected those who also completed the additional functional gastrointestinal disorder questionnaire in 2013 (n = 17,905). Among them, 2802 individuals who declared an organic disease or alarm symptoms were excluded. The sample included 15,103 participants, mainly women (73.4%), and the mean age in this population was 55.8 ± 13.2 years. The selection procedure is illustrated in Figure 1.

Overall, 812 (5.4%) participants were identified as having an IBS, and a higher prevalence in women was estimated (6.5 vs. 2.3%, *p* < 0.001). Prevalence for IBS subtypes in this sample was 1.0% for IBS-C, 1.6% for IBS-D, 2.3% for IBS-M, and 0.4% for IBS-U in the total sample.

In comparison with non-IBS individuals, participants with IBS were more likely to be younger, more educated, and less likely to be retired and to have a high physical activity (Table 1).

Daily intakes of macro and micronutrients were also compared between IBS and their non-IBS counterparts (Table S1). sPNNS-GS2, reflecting the nutritional quality of the diet, was not significantly different between IBS and non-IBS participants.

### 3.2. Gluten Avoidance among IBS and Non-IBS Participants

Participants with IBS were more likely to declare a gluten avoidance, either total or partial avoidance, than other participants (respectively 3.0% vs. 1.6% for total and 11.8% vs. 7.2% for partial, *p* < 0.0001). Prevalence for self-reported gluten intolerance or sensitivity was higher for participants with IBS than other participants (6.8% vs. 2.1%, *p* < 0.0001) of whom 29.1% and 29.4%, respectively, where medically diagnosed. Participants with IBS declared more often suffering from food allergies than other participants (12.9% vs. 8.1%, *p* < 0.0001) (Table 2).

Among gluten avoiders, the occurrence of physical symptoms is the main reason for avoiding gluten and is more frequently reported by IBS participants than by non-IBS participants (67.5% vs. 46.9%). Specifically, participants with IBS more frequently reported having a gluten-free diet for their physical comfort and well-being than other participants (43.3% vs. 34.7%), with the remainder being for health reasons as allergies and intolerances (24.2% vs. 12.2%). Besides, compared to IBS individuals, non-IBS reported more frequent long-term health for gluten avoidance (20.8% vs. 38.1%).

Overall, participants with IBS reported more food avoidances than other participants, both partial and total avoidance. Among these, 29.8% of IBS participants declared following a lactose-free diet (for health purposes) and only 18.8% for the group without IBS. In addition, among IBS participants, 9.2% reported both gluten and lactose avoidance, versus 5.5% for non-IBS participants.

### 3.3. Association between IBS and Gluten Avoidance

Associations between IBS and gluten avoidance are exhibited in Table 3. In models 1 and 2, total and partial gluten avoidance was positively associated with IBS, for total (OR = 1.86, 95%CI = 1.21, 2.85), and partial avoidance (OR = 1.71, 95%CI = 1.36, 2.14). Adjustment for BMI attenuated partially the association for total avoiders only. After adjustment for adherence to recommendations (model 4), gluten avoidance and particularly total avoidance were significantly associated with IBS, for total (OR = 1.83, 95%CI = 1.19, 2.83), and partial avoidance (OR = 1.73, 95%CI = 1.38, 2.17). Considering IBS subtypes, similar associations were observed for IBS-D (OR = 1.73, 95%CI = 1.19, 2.50) and IBS-M (OR = 1.82, 95%CI = 1.34, 2.47) (Table 4).

## 4. Discussion

In this cross-sectional study, we showed that IBS participants were more likely to report gluten avoidance, especially a total avoidance compared to non-IBS participants. Regarding IBS subtypes, gluten avoidance was strongly higher in IBS-D and IBS-M.

In the present study, among the general population, individuals with IBS were identified according to the Rome III criteria and the proportion of IBS was estimated to be 5.4%. It is well known that the vast majority of IBS are not medically diagnosed [31]. Indeed, one European study reported that more than half of undiagnosed IBS were misevaluated by primary care and few primary care referred patients to a gastroenterologist [32]. Besides, it was also much due to the lack of medical consultation under presumed GI symptoms [33]. This under-diagnosis probably leads to the implementation of voluntary behaviors to alleviate perceived symptoms [31].

In this population, the prevalence of gluten avoidance in IBS individuals was estimated at 14.8% and was significantly higher than in the overall cohort—estimated at around 9%.

In most studies, foods containing gluten, primarily wheat, were reported to worsen gastrointestinal (GI) symptoms in IBS patients [7,8,34,35] and these foods were more reduced or excluded in IBS individuals compared to non-IBS participants [36]. This is also consistent with our results about the reason for eviction, indicating that the proportion of individuals excluding gluten for physical comfort and well-being was higher for IBS participants than their counterparts (43.3% and 34.7%, respectively).

In line with previously documented studies, our participants with IBS declared more self-reported food avoidances and, notably, to a lesser extent, more self-reported food allergies compared to other participants [8,36]. In the literature, actual food allergy is rare in IBS and the general population, but perceived food intolerances partly explain food sensitivity in IBS patients [7,37,38]. Thus, differences between perceived and actual food intolerances are relevant to consider [34,36]. Nevertheless, a high number of perceived food intolerances increase the severity of GI symptoms and reduce the quality of life-related to diet [8]. This multiplicity of perceived food intolerances may encourage IBS patients to be more attentive to their food choices [39] and may result in reducing or avoiding certain foods in their diets [7,36].

Besides perceived symptoms, gluten has been shown to cause adverse reactions in IBS participants. Prospective, non-controlled, or double-blind placebo-controlled trials showed that a gluten avoidance has beneficial effects on GI symptoms in patients with IBS [40,41] and particularly with IBS-D [13,42,43]. In our study, IBS subjects also excluded gluten for health reasons (intolerance, allergy) more than other participants (24.2% vs. 12.2%). This is in line with the higher prevalence of self-reported gluten intolerance or sensitivity observed in our IBS population and the prevalence was estimated at 6.8% in our IBS participants compared to 2.1% in non-IBS patients.

Apart from recognized gluten-related disorders, non-celiac gluten sensitivity (NCGS) has been recently characterized by intestinal and extra-intestinal symptoms triggered by gluten ingestion and generally, NCGS is self-declared [44]. However, a meta-analysis revealed that more than 80% of patients presumed to have NCGS in clinical studies did not meet the Salerno experts’ criteria, recently defined as the gold standard to diagnose NCGS [45,46]. Even formally defined, the diagnosis is difficult to perform in clinical practice and then to estimate [47]. To date, the overlap between individuals with NCGS and IBS has been clearly established [44,48]. Both outcomes are difficult to distinguish correctly, while significant placebo and nocebo responses have been highlighted in IBS trials [49]. These effects occur among almost 40% of presumed NCGS, while only 16% could be considered gluten-specific [46].

Globally, IBS individuals are seeking foods to exclude to attenuate their symptoms. In the literature, between 62% and 94% of individuals with IBS have experimented with dietary interventions to alleviate symptoms [7,36] and most of them are without medical follow-up [7]. Also reported for the lactose-free diet, the gluten-free diet is significantly self-implemented as a diet modification in IBS [16], leading mostly to unsupervised diets [50]. In contrast, guidelines from the British Dietetic Association (BDA) and the National Institute of Clinical Excellence (NICE) only address general recommendations for a healthy diet and lifestyle for IBS patients [51,52]. Official guidelines briefly mention advanced dietary interventions and appear to be disconnected from patients preferences [5,53].

Misunderstandings between gluten and other wheat components, including FODMAPs in the intestinal disorders aetiologia, still remain. Along with gluten, a diet low in FODMAPs has been shown to improve IBS-like symptoms and has been ultimately recommended by practitioners and previous IBS guidelines cited [54,55]. To improve compliance [54], different approaches to a low-FODMAP diet have been suggested. Wheat, rich in fructans, is a major source of FODMAPs [56]. Highly favored by patients, a wheat-free or even gluten-free diet could be a strategy considering a “bottom-up” approach in the implementation of a low-FODMAP diet [54,57].

Regarding nutrient intakes, some studies showed similar results between IBS cases and those of the general population as observed in the present analysis [39,58], and sometimes even a less healthy diet [59]. In the present study, IBS participants did not exhibit particular low nutrient intakes, even with higher self-reported food avoidances. Nevertheless, in the literature, a gluten-free diet is not exempt from nutritional risks focusing on the overall restrictive dietary approach [50,60]. Even with reported high costs [61], gluten avoiders may find a convenient option in the rising market of gluten-free foods in substitution to their diet. In the present study, the mention of “gluten-free” on food products was not assessed in our dietary records. However, the substitution of gluten in food products requires modification to achieve similar characteristics and quality. Then, adding ingredients, additives, and process modification may convey adverse health effects (e.g., microbiota disruption suggested for several emulsifiers) [62], which supports associations between ultra-processed foods and higher prevalence of IBS [63]. Further studies need to explore the potential long-term impact of gluten-free ultra-processed foods.

The present study had some limitations. Due to the cross-sectional and observational design, causality was not assessed (i.e., if gluten avoidance tends to reduce IBS symptoms). In addition, even though it is a chronic disease, IBS is punctuated by relapses and remissions that may have occurred between the two questionnaires. Participants from the NutriNet-Santé cohort are volunteers and, thus, are probably more likely to be health-conscious. Thus, the external validity of this cross-sectional study may be affected as this population is not representative of the French population.

However, a high variability in the avoidance diet allowed us to detect differences in nutritional behaviors between IBS and other participants. Then, the onset of IBS was defined by a symptom-based evaluation established by the Rome III criteria and by the exclusion of reported digestive diseases or alarming symptoms. It should be noted that diagnosis of celiac disease, NCGS, and IBS are often confused, and no proper validation of cases was conducted. Nonetheless, at the time of inclusion, the Rome III criteria were considered as the gold standard to identify IBS [64], and the prevalence of IBS cases was similar to those reported in other French studies [65]. Food avoidances and health outcomes as gluten sensitivity were based on self-declaration. Regarding the outcome, participants’ declaration about their gluten avoidance allowed us to classify them into three groups: total, partial, or non-avoiders and no quantification of gluten intake was properly estimated. The start date for gluten avoidance was not assessed and could be used to evaluate the diet implementation compared to the identification of IBS.

Some strengths can be exhibited. To the best of our knowledge, this is the first cross-sectional observational study to compare the grade of self-reported gluten avoidance in IBS and non-IBS participants among a large adult population. As discussed above, a substantial proportion of IBS patients is not diagnosed, and most recent studies have been conducted on clinically diagnosed cases. The present study identified IBS cases using the Rome III criteria among the general population and described the practices observed among clinically diagnosed and undiagnosed IBS cases.

## 5. Clinical Implications

Although widely questioned, patients show very high adherence to the gluten-free diet, which could be a strategy to improve the patient acceptance of the low FODMAP diet during its implementation. Nevertheless, given the number of intolerances reported by people with IBS, it is clinically important to implement personalized dietary follow-up, supervised by a qualified dietitian. This is particularly relevant in the context of the increasing availability of processed gluten-free foods.

## 6. Conclusions

In conclusion, gluten avoidance is consciously followed by a substantial proportion of IBS patients as part of a higher overall dietary management. Globally misdiagnosed, IBS individuals probably alleviate their perceived symptoms by adopting self-implemented behaviors. Although mostly unjustified in the general population, gluten avoidance may likely be beneficial for a subset of IBS with NCGS. Overall, participants with IBS did not exhibit low nutrient intakes, even with higher food avoidances, but potential adverse effects related to food restriction and substitution may occur and require further research. Finally, gastrointestinal disorders must be properly recognized, diagnosed, and managed under medical supervision. In addition to appropriate guidelines, the supervision of a qualified dietician is strongly encouraged in IBS patients for a personalized dietary framework.

## Figures and Tables

**Table 1:** Sociodemographic and lifestyle characteristics according to IBS by French adults (NutriNet-Santé study, 2016, *n* = 15,103).

	IBS	No IBS		Total
	*n* = 812	*n* = 14,291	*p* ^a^	*n* = 15,103
	%	%		%
**Sex**			<0.0001	
Women	88.4	72.5		73.4
Men	11.6	27.5		26.6
**Age** (years)			<0.0001	
*Mean*	52.5	56.0		55.8
*SD*	13.5	13.2		13.2
**Age Class** (%)			<0.0001	
<25	0.3	0.4		0.4
25–39	17.7	12.0		12.3
40–54	28.9	23.6		23.8
55–64	24.8	25.6		25.6
≥65	28.3	38.5		37.9
**Educational Level** (%)			0.0016	
No diploma or primary	11.3	14.7		14.6
Secondary	16.3	17.8		17.7
Higher education	72.4	67.4		67.7
**Occupational Category** (%)			<0.0001	
Farmer	0.3	0.3		0.3
Self-employed	1.4	1.2		1.2
Employee	12.0	10.0		10.1
Manual worker	0.3	0.6		0.6
Intermediate profession	16.1	13.6		13.8
Managerial staff, intellectual profession	24.0	21.0		21.1
Unemployed	11.7	8.5		8.7
Student	0.7	0.7		0.7
Retired	33.6	44.3		43.7
**Monthly Income Per Household Unit** (%)			0.8702	
<1200 €	7.4	6.7		6.7
1200–2300 €	34.5	33.9		34.0
>2300 €	44.3	47.0		46.9
Refuse to declare	13.8	12.4		12.5
**Marital Status** (%)			0.3105	
Single, separated or widowed	25.6	24.1		24.1
As a couple or married	74.4	76.0		75.9
**Size of the Urban Residence Unit** (%)			0.0034	
Rural	20.2	22.3		22.2
<20,000 inhabitants	14.9	15.9		15.8
20,000–200,000 inhabitants	17.1	18.1		18.0
>200,000 inhabitants	47.8	43.8		44.0
**Smoking Status** (%)			0.0166	
Smoker	10.1	7.4		7.6
Former smoker	40.0	40.2		40.2
Non-smoker	49.9	52.4		52.3
**BMI**			0.0005	
*Mean*	23.2	23.7		23.7
*SD*	4.3	4.0		4.0
**BMI Class** (%)			0.0305	
Underweight	4.3	2.6		2.7
Normal	69.2	67.6		67.7
Overweight	19.5	22.9		22.7
Obesity	7.0	7.0		7.0
**Physical Activity** (%)			0.0053	
Low	22.9	20.9		21.0
Moderate	43.1	39.6		39.7
High	34.0	39.6		39.3

Abbreviations: IBS, Irritable Bowel Syndrome; BMI, Body Mass Index; SD, Standard Deviation. ^a^ Values are % except otherwise is specified, *p*-value referred to chi^2^ test or ANOVA.

**Table 2:** Self-reported food avoidances and allergies according to IBS status (NutriNet-Santé study, 2016, *n* = 15,103).

	IBS	No IBS		Total
	*n* = 812	*n* = 14,291	*p* ^a^	*n* = 15,103
	%	%		%
**Type of Gluten Avoidance** (%)			<0.0001	
Total avoiders	3.0	1.6		1.7
Partial avoiders	11.8	7.2		7.4
Non-avoiders	85.2	91.2		90.9
**Motives for Gluten Avoidance** (among gluten avoiders) (%)			<0.0001	
Allergy, intolerance	24.2	12.2		14.5
Physical comfort and well-being	43.3	34.7		38.8
Price	0.8	1.0		1.0
Taste	6.7	11.2		11.8
Belief in a long-term health impact	20.8	38.1		40.1
Environmental reasons	1.7	1.6		1.7
Ethics reasons: respect for labour and human rights in production, fair trade	0.8	0.8		0.9
Convenience	1.7	0.5		0.6
**Gluten Intolerance or Sensitivity** (%)	6.8	2.1	<0.0001	2.3
**Lactose Avoidance for Health Reasons** (%)	29.8	18.8	<0.0001	19.4
**Lactose and Gluten Avoidance** (%)	9.2	5.5	<0.0001	5.7
**Number of Self-Reported Total or Partial Food Avoidances**			<0.0001	
*Mean*	16.8	14.2		14.3
*SD*	11.3	10.2		10.3
**Number of Self-Reported Total Food Avoidances**			<0.0001	
*Mean*	6.9	5.9		5.9
*SD*	5.8	5.4		5.5
**Number of self-Reported Partial Food Avoidances**			<0.0001	
*Mean*	10	8.3		8.4
*SD*	7.9	7.1		7.2
**Individuals Self-Reported Suffering from Food Allergies** (%)	12.9	8.1	<0.0001	8.4
**Number of Self-Reported Allergies** (among allergic sufferers)			<0.0001	
*Mean*	1.6	1.2		1.3
*SD*	1.3	0.9		1

Abbreviations: IBS, Irritable Bowel Syndrome; BMI, Body Mass Index; SD, Standard Deviation. ^a^ Values are % except otherwise is specified, *p*-value referred to chi^2^ test or ANOVA.

**Table 3:** Adjusted associations between gluten avoidance and IBS (NutriNet-Santé study, 2016, *n* = 15,103) ^a^.

	Non-Avoiders	Total Avoiders		Partial Avoiders		
	*n* = 13,723	*n* = 256		*n* = 1124		*p* ^b^
		OR	95%CI	OR	95%CI	
Model 1 ^c^	Ref.	1.86	(1.21, 2.85)	1.71	(1.36, 2.14)	<0.0001
Model 2 ^d^	Ref.	1.88	(1.22, 2.89)	1.73	(1.38, 2.17)	<0.0001
Model 3 ^e^	Ref.	1.82	(1.18, 2.81)	1.73	(1.38, 2.16)	<0.0001
Model 4 ^f^	Ref.	1.83	(1.19, 2.83)	1.73	(1.38, 2.17)	<0.0001
Model 5 ^g^	Ref.	1.88	(1.21, 2.94)	1.75	(1.39, 2.20)	<0.0001

Abbreviations: IBS, Irritable Bowel Syndrome; PCA, principal component analysis; sPNNS-GS2, simplified Programme National Nutrition Santé-Guidelines Score 2; OR, Odd-ratio; 95%CI, 95% confidence interval; BMI, Body Mass Index. ^a^ Values are odd-ratio and 95% confidence interval estimated using multinomial logistic regression. ^b^ *p*-value obtained with multinomial logistic regression using gluten avoidance as a categorical variable. ^c^ Model 1: adjusted on sex and age (continuous). ^d^ Model 2: adjusted on sex, age (continuous), educational level, monthly household income, occupational category, physical activity, smoking status, total energy intake excluding alcohol (continuous), alcohol (continuous) and the number of dietary records filled in since the inclusion (continuous). ^e^ Model 3: Model 2 + BMI (continuous). ^f^ Model 4: Model 3 + sPNNS-GS2 (continuous). ^g^ Model 5: Model 3 + Dietary patterns (PCA factors) (continuous).

**Table 4:** Associations between gluten avoidance and IBS subtypes (NutriNet-Santé study, 2016, *n* = 15,103) ^a^.

Model	Total or Partial Avoiders vs. Non-Avoiders *n* = 1380			*p* ^b^
	*n*	OR	95%CI	
**IBS subtypes: Model 4 ^c^**				<0.0001
No IBS	1260	1	Ref.	
IBS-C (vs. No IBS)	20	1.44	(0.89, 2.33)	
IBS-D (vs. No IBS)	35	1.73	(1.19, 2.50)	
IBS-M (vs. No IBS)	52	1.82	(1.34, 2.47)	
IBS-U (vs. No IBS)	13	2.30	(1.23, 4.30)	

Abbreviations: IBS, Irritable Bowel Syndrome; IBS-C, IBS with predominant constipation; IBS-D, IBS with predominant diarrhea; IBS-M, IBS with predominant irregular bowel habits (mixed C/D); IBS-U, IBS unclassified; OR, Odd-ratio; 95%CI, 95% confidence interval; BMI, Body Mass Index; sPNNS-GS2, simplified Programme National Nutrition Santé-Guidelines Score 2. ^a^ Values are Odd-ratio and 95% confidence interval estimated using binomial logistic regression. ^b^ *p*-value obtained with binomial logistic regression using gluten avoidance as a categorical variable. ^c^ Model 4: adjusted on sex, age (continuous), educational level, monthly household income, occupational category, physical activity, smoking status, total energy intake excluding alcohol (continuous), alcohol (continuous) and the number of dietary records filled in since the inclusion (continuous)+ BMI (continuous) + sPNNS-GS2 (continuous).

## Data Availability

Data used in this study are under the protection of national health data regulations set forth by the French National Commission on Informatics and Liberty (Commission Nationale de l’Informatique et des Libertés, CNIL), which prohibit free public access. The data can be made available upon written request sent to the NutriNet-Santé operational coordinator, Nathalie Druesne-Pecollo (n.pecollo@eren.smbh.univ-paris13.fr), and following approval by the NutriNet-Santé steering committee.

## Supplementary Materials

The following are available online at https://www.mdpi.com/article/10.3390/nu13114147/s1. Table S1. Daily intake of macronutrients and micronutrients according to IBS status (NutriNet-Santé study, 2016, *n* = 15,103); Table S2. Dietary patterns obtained by factor analysis using principal component analysis of daily food intakes (NutriNet-Santé study, 2016, *n* = 15,103).
