# Supplementary material for: Association between Self-Reported Gluten Avoidance and Irritable Bowel Syndrome: Findings of the NutriNet-Santé Study
Source: Nutrients. 2021 Nov 19;13(11):4147. doi: 10.3390/nu13114147 (PMC8622067; doi:10.3390/nu13114147)
Supplement: Supplementary file 1 [file nutrients-13-04147-s001.zip › nutrients-1471219-supplementary.pdf]

Table S1. Daily intake of macronutrients and micronutrients according to IBS status (NutriNet-Santé study, 2016, n=15,103).

	**IBS (> 6 months)**		**No IBS**			**Total**	
	n=812		*n=14,291*		p[a]	*n=15,103*	
	Mean	SD	Mean	SD		Mean	SD
**Adequacy score to PNNS4 guidelines (sPNNSGS2)** (/ 14.25)	1.8	3.2	1.6	3.4	0.0515	1.6	3.4
**Total energy intake excluding alcohol** (kcal/d)	1809.7	338.8	1860.8	403.7	0.0004	1858.1	400.6
**Alcohol** (g/d)	7.5	9.7	8.7	11.3	0.003	8.6	11.3
**Percentage of total energy intake excluding alcohol from:**							
**Carbohydrates** (%)	42.5	5.9	42.3	5.9	0.567	42.4	5.9
**Lipids** (%)	40.9	5.6	40.7	5.6	0.169	40.7	5.6
**Omega 3**[b] (g/d)	1.5	0.7	1.5	0.8	0.477	1.5	0.7
**Omega 6**[b] (g/d)	10.0	3.1	9.8	3.3	0.194	9.8	3.3
**Proteins** (%)	16.3	2.8	16.7	2.9	0.0002	16.7	2.9
**Plant proteins / Total protein intake** (%)	35.5	11.1	34.6	11.1	0.020	34.6	11.1
**Fibres**[b] (g/d)	20.8	5.9	20.9	5.8	0.445	20.9	5.8
**Iron**[b] (mg/d)	13.8	3.7	13.9	3.8	0.451	13.9	3.8
**Zinc**[b] (mg/d)	10.3	2.1	10.6	2.2	0.003	10.6	2.2
**β-carotene**[b] (mg/d)	3.7	2.1	3.6	2.1	0.859	3.6	2.1
**Vitamin B9**[b] (µg/d)	318.2	80.9	327.1	83.2	0.003	326.7	83.1
**Vitamin B12**[b] (µg/d)	5.0	3.8	5.3	4.1	0.012	5.3	4.1
**Vitamin C**[b] (mg/d)	105.6	48.5	111.9	63.5	0.005	111.5	62.8
**Vitamin E**[b] (mg/d)	12.4	3.6	12.2	3.6	0.224	12.2	3.6
**Number of dietary records completed**	8.2	3.7	8.3	3.5	0.501	8.3	3.5

Abbreviations: IBS, Irritable Bowel Syndrome ; SD, Standard Deviation; PNNS4, Programme National Nutrition Santé 4; sPNNS-GS2, simplified Programme National Nutrition Santé-Guidelines Score 2

[a]p-value of the type III ANOVA for quantitative variables

[b]Nutrient intakes are adjusted for energy using the residual method.

Table S2. Dietary patterns obtained by factor analysis using principal component analysis of daily food intakes (NutriNet-Santé study, 2016, n=15,103)[a].

	Healthy diet	Traditional diet	Western diet
**Vegetables**	0.626	0.400	-0.007
**Fruits**	0.516	0.198	0.087
**Dried fruits**	0.339	-0.049	0.099
**Meat**	-0.371	0.293	0.029
**Poultry**	-0.074	0.070	0.045
**Offal**	-0.062	0.222	0.066
**Processed meat**	-0.426	0.223	0.306
**Fish and seafood**	0.168	0.167	0.157
**Eggs**	0.164	0.124	0.121
**Milk and dairy products**	-0.082	0.042	-0.494
**Cheese**	-0.199	0.294	0.368
**Refined starchy food**	-0.037	-0.029	0.175
**Bread**	-0.413	0.497	-0.067
**Potatoes and other tubers**	-0.028	0.542	-0.010
**Legumes**	0.343	0.010	0.163
**Whole starchy food**	0.549	-0.071	0.173
**Added fats**	-0.079	0.551	-0.312
**Oils**	0.297	0.217	0.415
**Sauce**	0.268	0.518	-0.393
**Salty or sweet, fatty products**	-0.284	-0.111	0.121
**Sugar-free alcohol-free beverages**	0.279	0.004	0.217
**Sweet beverages**	-0.253	-0.151	0.060
**Alcoholic beverages**	-0.312	0.242	0.531

[a]Values are factor-loadings
